# Cardiovascular magnetic resonance imaging in patients with MR-conditional devices: a UK tertiary centre experience on image quality and change in patients management

**DOI:** 10.1186/1532-429X-17-S1-P238

**Published:** 2015-02-03

**Authors:** Nauman Ahmed, Amardeep Ghosh Dastidar, Elisa McAlindon, Chris B Lawton, Daniel Augustine, Glyn Thomas, Edward Duncan, Tim Cripps, Ihab Diab, Chiara Bucciarelli-Ducci

**Affiliations:** Cardiology, 1Bristol Heart Institute, Bristol NIHR Cardiovascular Biomedical Research Unit (BRU), Bristol, United Kingdom, Bristol, UK

## Background

Cardiovascular magnetic resonance (CMR) examinations is increasingly used in daily clinical practice. Conventional pacemakers and implantable cardioverter-defibrillators (ICD) have always been regarded as a contraindication to MR imaging. However the introduction of MR-conditional systems have significantly improved access to MR examinations. Limited data exists regarding indications and management outcomes and impact of different cardiac rhythm devices on image quality

## Methods

Between June 2012 to September 2014 we identified 34 consecutive patients with cardiac rhythm devices who were referred for a CMR examination in our tertiary cardiothoracic centre. All devices were interrogated pre-CMR and post-CMR to minimize interference with the electromagnetic fields and in case reprogrammed after the CMR. All scans were performed on 1.5 Tesla with eight-channel phased-array receiver coils. The indication of CMR and impact of CMR findings on patient management was also recorded.

## Results

Among the 34 patients with cardiac devices undergoing a CMR, 16(47%) had pacemakers(15 left sided and 1 right sided) and 18(53%) implantable loop recorder (ILR). All pacemakers scanned were MR conditional. In the post-CMR interrogation, there were no significant change of pacing capture threshold, lead impedance and battery life. Indications and CMR data resulting in new diagnosis and impact on management shown in Table 1. Artefact due to the cardiac device was identified in 15/34 (44%) of the scans, and no artefacts in 19(56%) patients. Artefacts were assessed in the cine and late gadolinium enhancement image and categorized into minor artefacts (n=15) and major artefacts (n=2), the latter group providing major limitation to the diagnostic accuracy of the CMR scan. Among the 15 devices providing minor artefacts, n=2 were pacemakers vs n=13 ILR (p<0.001). Of those 2 providing major artefact 1 was a pacemaker and 1 was a ILR (p=NS). Of the devices not causing artefacts (n=19), 13 were pacemakers and 6 ILR. Cine SSFP and gadolinium imaging sequences were performed in all patients with additional FLASH sequences performed in the two patients with major artefacts. Overall, the SSFP cine was the sequence most commonly affected by artefact even leading to non-diagnostic images in 2 patients. Gadolinium sequences were non diagnostic in 1 examination (in one of the 2 patients where major artefacts seen).

**Table 1:** Table 1

Indication	n= 34(%)	Diagnosis by CMR n=15 (44%)	CMR Impact on management n=(34%)
Infiltrative disease (amyloidosis/ sarcoidosis)	5 (15)	3	3
Possible Cardiomyopathy	7 (20)	5	2
Assessment of LV function	1 (3)	1	1
Aetiology for syncope & AV block	11 (32)	4	2
Ischaemia/Viability	4 (12)	-	2
Iron over loading	1 (3)	1	1
Pericardial disease	1 (3)	0	0
Aortic pathology	1(3)	1	1
Congenital heart disease	3 (9)	-	-

Overall, CMR established a new diagnosis in 44% of the patients, and provided additional information with impact on clinical management in 34% of the patients.

## Conclusions

CMR can be performed safely in patients with ILR and MR conditional pacemakers with strictly defined cardiologic and radiologic protocols and monitoring. Most of the devices, particularly ILR, can cause artefacts but the interference with the diagnostic accuracy of the CMR scan is only minor. In patient with MR-conditional device, CMR can provide important diagnostic information that can impact management.

## Funding

None.

